# Quantitative Third Harmonic Generation Microscopy for Assessment of Glioma in Human Brain Tissue

**DOI:** 10.1002/advs.201900163

**Published:** 2019-04-05

**Authors:** Zhiqing Zhang, Jan C. de Munck, Niels Verburg, Annemieke J. Rozemuller, Willem Vreuls, Pinar Cakmak, Laura M. G. van Huizen, Sander Idema, Eleonora Aronica, Philip C. de Witt Hamer, Pieter Wesseling, Marie Louise Groot

**Affiliations:** ^1^ LaserLab Amsterdam Department of Physics and Astronomy Faculty of Sciences Vrije Universiteit Amsterdam De Boelelaan 1081 1081 HV Amsterdam The Netherlands; ^2^ Department of Radiology and Nuclear Medicine Amsterdam Universities Medical Center/VU University Medical Center De Boelelaan 1118 1081 HZ Amsterdam The Netherlands; ^3^ Amsterdam Neuroscience Vrije Universiteit Amsterdam De Boelelaan 1085 1081 HV Amsterdam The Netherlands; ^4^ Amsterdam Brain Tumor Center Amsterdam Universities Medical Center/VU University Medical Center De Boelelaan 1117 1081 HV Amsterdam The Netherlands; ^5^ Department of Neurosurgery Amsterdam Universities Medical Center/VU University Medical Center De Boelelaan 1117 1081 HV Amsterdam The Netherlands; ^6^ Department of Pathology Amsterdam Universities Medical Center/VU University Medical Center De Boelelaan 1117 1081 HV Amsterdam The Netherlands; ^7^ Department of Pathology Canisius Wilhelmina Ziekenhuis Weg door jonkerbos 100, Postbus 9015 6500 GS Nijmegen The Netherlands; ^8^ Department of Pathology Gazi University Medical Faculty Besevler 06500 Ankara Turkey; ^9^ Department of (Neuro) Pathology Amsterdam UMC University of Amsterdam Meibergdreef 9 1105 AZ Amsterdam The Netherlands; ^10^ Princess Máxima Center for Pediatric Oncology and Department of Pathology University Medical Center Utrecht Heidelberglaan 25 3584 CS Utrecht The Netherlands

**Keywords:** glioma infiltration, label‐free microscopy, neuropathology, neurosurgery, third harmonic generation

## Abstract

Distinguishing tumors from normal brain cells is important but challenging in glioma surgery due to the lack of clear interfaces between the two. The ability of label‐free third harmonic generation (THG) microscopy in combination with automated image analysis to quantitatively detect glioma infiltration in fresh, unprocessed tissue in real time is assessed. The THG images reveal increased cellularity in grades II–IV glioma samples from 23 patients, as confirmed by subsequent hematoxylin and eosin histology. An automated image quantification workflow is presented for quantitative assessment of the imaged cellularity as a reflection of the degree of glioma invasion. The cellularity is validated in three ways: 1) Quantitative comparison of THG imaging with fluorescence microscopy of nucleus‐stained samples demonstrates that THG reflects the true tissue cellularity. 2) Thresholding of THG cellularity differentiates normal brain from glioma infiltration, with 96.6% sensitivity and 95.5% specificity, in nearly perfect (93%) agreement with pathologists. 3) In one patient, a good correlation between THG cellularity and preoperative magnetic resonance and positron emission tomography imaging is demonstrated. In conclusion, quantitative real‐time THG microscopy accurately assesses glioma infiltration in ex vivo human brain samples, and therefore holds strong potential for improving the accuracy of surgical resection.

## 1 Introduction

Originating from (precursors of) glial cells, glial tumors (gliomas) account for ≈80% of primary brain tumors.^1^ The vast majority of gliomas are so‐called “diffuse gliomas” because they extensively infiltrate into the surrounding normal brain. Especially in high‐grade malignant gliomas, the tumor generally shows a gradient of invasive tumor cells from the highly cellular central tumor mass toward the noninvaded normal tissue.^2^, ^3^ Multimodal treatment of diffuse gliomas involves surgery, radiotherapy, and chemotherapy.^4^ The aim of a resection is to maximize removal of tumor while preserving the patient's functional performance.^5^, ^6^ More tumor removal is associated with longer survival, however loss of function from too extensive resection is associated with lower quality of life and shorter survival.^7^ Even after radiologically complete resection of the contrast‐enhancing part of glioblastoma, local recurrence occurs in 85% of patients.^8^ Thus there is at present ample room for improvement of the prognosis and survival rate of patients with diffuse gliomas.^1^, ^9^

The lack of a well‐defined biological interface between diffuse glioma and normal brain tissue makes intraoperative recognition of tumor boundaries by neurosurgeons difficult. Shortcomings of clinical imaging techniques such as magnetic resonance imaging (MRI)/intraoperative MRI, intraoperative computed tomography (CT), ultrasound, and bright field neurosurgical microscopes include too low resolution for detection of invasive tumor cells at the microscopic level, quality of staining, and/or lack of real‐time imaging.^10^, ^11^, ^12^ Consequently, their contribution to surgical outcomes has been questioned.^13^, ^14^

The availability of a tool allowing for intraoperative detection of not only the highly cellular central glioma mass, but also the (gradient of) more peripherally located invasive tumor cells can be expected to contribute to improved surgical resection of these tumors. Indeed, great effort has recently been put into detection of invasive glioma cells at (sub)cellular resolution. Fluorescence‐guided surgery has been shown to improve extent of resection in high‐grade glioma resection but it appears to be difficult to detect low‐grade/low‐cellularity glioma infiltration.^15^, ^16^ In contrast to the fluorescence techniques, label‐free optical techniques have the advantages of high resolution and tissue preparation and staining is not required. Swept‐source optical coherence tomography (OCT) has been shown to allow for discrimination of normal brain tissue from areas infiltrated by glioma cells in ex vivo samples based on optical attenuation differences.^17^ However, the OCT techniques reveal little or no cellular information, which may limit its sensitivity for detection of glioma infiltration. Raman techniques such as Raman spectroscopy, coherent anti‐Stokes Raman scattering microscopy, and stimulated Raman scattering microscopy have been particularly promising in the detection of glioma infiltration indicated by cellularity in ex vivo human brain specimens or in vivo patients’ brain.^18^, ^19^, ^20^, ^21^, ^22^ However, in order to enable detection of the spectral differences between glioma infiltration and normal brain tissue, Raman spectroscopic techniques need extensive comparison of the acquired spectra with libraries of reference spectra. The spectral differences revealed are only subtle which may result in a lower sensitivity and less adequate detection of glioma infiltration, especially in areas with a limited number of tumor cells. For example, a sensitivity limit of 17 tumor cells/150 × 150 µm^2^ was reported for Raman spectroscopy.^18^

Third and second harmonic generation (THG/SHG) microscopy differs from OCT and Raman spectroscopy in the sense that it directly visualizes cellular morphology in brain tissue, and in comparison to Raman microscopies, it is technically much simpler.^23^, ^24^, ^25^, ^26^, ^27^ We have previously demonstrated how this tool enables visualization of cellular morphology in normal mouse brain ex vivo and in vivo.^26^ We have also further reported that THG directly visualizes important histopathological hallmarks of diffuse gliomas (increased cellularity, nuclear pleomorphism, and rarefaction of neuropil) in fresh, unprocessed human brain tissue samples,^28^ but a quantitative assessment of the presence/absence of glioma infiltration by THG is still lacking. As THG microscopy has the potential to directly reveal microscopic details of the tissue, it may represent a very promising tool for real‐time ex vivo and, ultimately, in situ assessment of (the border of) diffuse gliomas, as well as for improved management of patients with, e.g., cancer of the skin or breast.^29^, ^30^

Here, we test the feasibility of THG microscopy to detect brain tumor infiltration indicated by cellularity, in ex vivo fresh, unprocessed tissue samples from 23 patients undergoing surgical resection for a diffuse glioma or epilepsy. We demonstrate automated image analysis of the THG images to quantitate the contained information and to provide a fast, THG‐based diagnosis. We provide both qualitative and quantitative evidence demonstrating that the automated analysis of the THG images reflects the true cellularity of histologically normal brain and glioma tissue, and thereby demonstrate that THG microscopy holds great potential as a tool for ex vivo and, ultimately, in situ assessment of diffuse glioma infiltration in human brain.

## 2 Results

### 2.1 Qualitative Comparison of THG Imaging and Histology of Normal Brain and Diffuse Gliomas

Cellular morphology is a cornerstone for discriminating normal brain tissue from glioma tissue. We first extended our previous study on THG imaging of glioma tissue samples,^28^ by providing a more detailed interpretation of the cellular morphology observed with THG. We performed THG imaging on 56 biopsies from 23 patients with grades II–IV diffuse glioma or epilepsy (Table S1, Supporting Information).

**Figure 1:**
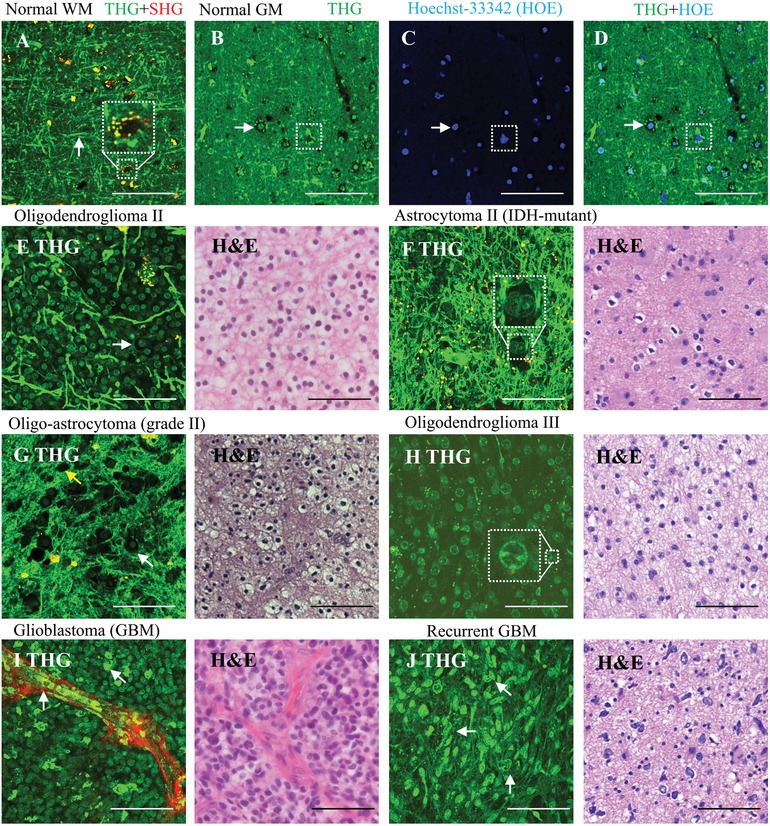
THG imaging of cellular morphology in normal brain and grades II–IV diffuse gliomas. Scale bar: 100 µm. THG/SHG/combined signals are shown in green/red/yellow, with image acquisition time of 8 s. Each THG image represents an optical slice of 2 µm, and the thickness of the H&E stained histological slides is 3 µm. A) THG imaging of normal white matter (WM), showing brain cells appearing as dark holes with lipid‐rich lipofuscin granules inclusion (square), and axons appearing as linear, bright fibers (arrow). B–D) Colocating THG and HOE fluorescence images of normal gray matter (GM) showing different morphologies of brain cells, e.g., dark holes with visible nuclei inside (arrow) and dark holes with lipid‐rich lipofuscin granules (square). E) Both THG and H&E images of low‐grade oligodendroglioma (WHO grade II) tissue from patient 5, show hypercellularity with the tumor cells (arrow) having a round nucleus and a “fried egg” appearance in the H&E image, the tumor cells causing axonal disruption. F) THG/H&E images of astrocytoma grade II from patient 19 reveals atypical‐appearing astrocytes marked by enlargement and pleomorphism of tumor cell nuclei (square). G) THG/H&E images of diffuse glioma with oligoastrocytoma phenotype from patient 4, showing a moderate cell density with an admixture of cells with more oligodendrocyte‐ (white arrow) or astrocyte‐like morphology (yellow arrow). H) THG/H&E images of anaplastic oligodendroglioma (WHO grade III) from patient 15, showing hypercellularity, nuclear enlargement (compare (E)), and multiple nucleoli (square). I) THG/H&E images of glioblastoma (patient 10), showing hypercellularity, vascular proliferative changes, and aggregation of macrophages (arrow). J) THG/H&E images of a recurrent glioblastoma (patient 18), with marked hypercellularity and prominent nuclear pleomorphism (arrow) in a fibrillary background. Note that cell/nucleus shape and intensity vary from (A) to (J) that reflect (disease) state of the cell, and preliminary findings indicate that bright nuclei may be condensation of chromatin (unpublished data).

Ten histologically normal tissue samples were obtained from seven patients undergoing temporal lobe resection for intractable epilepsy. Lipid‐rich components of brain tissue, e.g., the lipid‐rich myelinated axons in the neuropil (i.e., the dense network of neuronal and glial cell processes), are a major source of contrast for THG,^24^, ^26^ which results in most brain cells appearing as “dark holes” in the THG images of normal brain (**Figure** **1**A) due to the lack of optical interfaces of the cell bodies. Especially the lipid‐rich myelinated axons in the neuropil appear as bright fibers (Figure 1A). THG images of white matter and gray matter clearly differ from each other with regard to the aspect of neuropil and cellular constituents (Figure 1A,B). SHG signals with partial autofluorescence signals integrated, provide information complementary to THG, e.g., on blood vessels and lipofuscin granules (Figure 1). To demonstrate that THG visualizes almost all cells, Hoechst‐33342 (HOE) was used to label cell nuclei in normal gray matter,^31^, ^32^ and THG and three‐photon fluorescence images were acquired simultaneously and overlaid (Figure 1B,D). The overlay images show that brain cells as identified by THG microscopy can appear as a “dark hole,” a dark hole with a relatively bright nucleus inside or a dark hole with scattered lipofuscin granules (Figure 1B). Manual cell/nucleus counting of five overlaid images showed that 95.7% of the HOE labeled nuclei had corresponding brain cells as identified in THG images with high contrast. This high correspondence is in line with our previous study in which ≈90% of mouse brain cells labeled by HOE were indeed detected in THG.^33^

We next investigated the appearance of cellular morphology and density in THG images of grades II–IV gliomas. According to the 2016 WHO Classification of Tumors of the Central Nervous System, the vast majority of diffuse gliomas in adults are diffuse astrocytic tumors of different molecular classes (IDH‐wildtype or IDH‐mutant) and different malignancy grades (WHO grades II–IV), and low‐grade and anaplastic (WHO grades II and III) oligodendrogliomas.^34^, ^35^ Histologically low‐grade diffuse gliomas (WHO grade II) are mainly characterized by increased cellularity.^36^, ^37^ In oligodendroglioma WHO grade II tissue, the THG images reveal a high density of cell nuclei, observed as small spherical features surrounded by “dark” cytoplasm (Figure 1E) and a disruption of axons (compare Figure 1A). This morphological information revealed by THG is in agreement with the hypercellularity that is often found in histologically low‐grade oligodendrogliomas and with the “fried‐egg” appearance of the tumor cells (round nucleus surrounded by clear halo of cytoplasm in hematoxylin and eosin (H&E) stained images). THG images of astrocytoma WHO grade II (IDH1‐mutant without 1p19q codeletion) tissue reveal atypical‐appearing astrocytes with variable pleomorphism and enlargement of nuclei, which is confirmed in the H&E images of this tissue (Figure 1F). THG images of a tumor with a histologically low‐grade, mixed (“oligo‐astrocytoma”) phenotype reveal moderate hypercellularity with different cell shapes, partly more oligodendroglial, partly more astrocytic in phenotype (Figure 1G).

THG imaging was also able to reveal microvascular changes, one of the main histopathological hallmarks of diffuse gliomas of high grade malignancy (WHO grades III or IV).^36^, ^37^ THG images of oligodendroglioma WHO grade III tissue (Figure 1H) show nuclear enlargement with uniform morphology of cell nuclei, with some nuclei showing multiple nucleoli, as seen in the H&E images of this tissue. Furthermore, microvascular proliferation and tumor‐associated macrophages are observed in glioblastomas with THG microscopy and confirmed by H&E (Figure 1I). Moderate hypercellularity and tumor cells with prominent irregular shapes in a fibrillary background are observed in a recurrent glioblastoma tissue by both THG imaging and H&E (Figure 1J). Thus, THG images reveal much of the relevant diagnostic morphology of different glioblastoma types.

### 2.2 Quantitative Comparison of THG Imaging and Histology of Normal Brain and Diffuse Glioma

**Figure 2:**
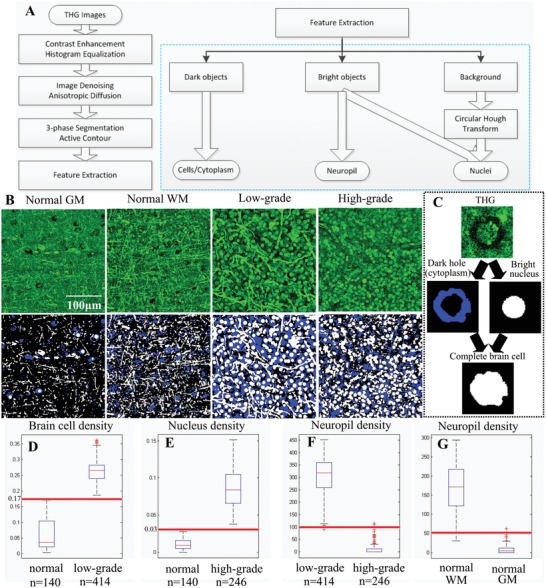
THG image quantification and quantitative difference among representative THG images of normal brain, histologically low‐grade, and high‐grade diffuse glioma tissues. We used the percentage of space (PoS) to represent the density of brain cells and nuclei. The density of the neuropil was measured by the perimeter of all detected neuropil. A) A general overview of the proposed quantification workflow. B) A THG image segmented into dark holes (blue), bright objects (white), and a background (black). C) Dark holes were combined with bright nuclei for full detections of brain cells. D) The density of brain cells in low‐grade glioma was higher than that in normal brain. E) The density of nuclei in high‐grade glioma was higher than that in normal brain. F) The density of neuropil in low‐grade glioma was higher than that in high‐grade glioma. G) The density of neuropil in normal white matter was higher than that in normal gray matter.

Based on these findings, demonstrating a very good “histology‐grade” (and even “cytology‐grade”) correlation between the morphology of diffuse gliomas in H&E and THG images, we proceeded with the development of an automated workflow for THG images to quantitatively assess the differences in cell density between histologically normal brain and glioma tissue. We have reported previously that the quantification of THG images is hampered by the information complexity.^38^ In order to tackle this problem, we have developed a 3‐phase segmentation algorithm to detect the “dark holes” and “bright” objects in a dim background, and tested the algorithm on THG images of normal brain tissue achieving an ≈99% segmentation accuracy.^38^ In this study, we incorporate the algorithm into an integrated quantification workflow (**Figure** **2**A; the Experimental Section) for processing THG images of normal brain and glioma tissue uniformly, enabling automated estimation of cell density and neuropil density. The challenge is to extract and separate the rich morphologies present in THG images. All THG images were first enhanced, denoised, and segmented into dark holes, bright objects, and a remaining background (Figure 2B). A dark hole represents either an entire brain cell, or the cytoplasm of a brain cell having a relatively bright nucleus inside, or a cell having lipofuscin granules in the cytoplasm (see Figure 1A,B). Bright objects consist of cell nuclei, neuropil, and lipofuscin granules. Object sphericity was used to select nuclei (sphericity > 0.5) and fiber‐like neuropil (sphericity < 0.1) for further statistical analysis.^39^ To obtain the correct measure of a brain cell, a dark hole/cytoplasm is combined with a bright nucleus if present (Figure 2C). To evaluate the segmentation accuracy, an additional software tool was developed to overlay the segmentation results with the raw THG images and to check if each object was correctly segmented. The automated workflow was able to detect the brain cells, nuclei, and neuropil present in THG images of both histologically normal and tumor tissue and the automated counting correlated well with manual methods (Figure S1 and Table S2, Supporting Information).

We then used this automated workflow to quantitative differences between THG images of normal brain and glioma tissue based on the differences in the measured density of brain cells, nuclei, and neuropil. We performed statistical analysis of the density of these features in 800 THG images obtained from a first group of patients, patients 1–10.^28^ We represented the density of brain cells/nuclei by the percentage of space (PoS) taken by brain cells/nuclei (see the Experimental Section), because in some tumor areas, the number of cells/nuclei was extremely high and their clumps were impossible to separate, indicating that PoS was an adequate representation for the density of brain cells/nuclei. The density of neuropil was quantified by the perimeter (in pixels) of all detected neuropil divided by 100. Statistical analysis showed that typical THG images of normal brain tissue, of histologically low‐grade, and of high‐grade glioma were significantly different, and the difference between one and another was revealed by one single feature (Figure 2D–G). The density of brain cells in normal brain (gray matter and white matter) for these cases was significantly lower than that of histologically low‐grade glioma tissues (Figure 2D). The nucleus density of the high‐grade glioma images was higher than that shown in THG images of normal (gray matter & white matter) brain (Figure 2E). The densities of neuropil obtained in THG images of histologically low‐grade glioma tissue and normal white matter were higher than that obtained in THG images of high‐grade glioma tissue and normal gray matter, respectively (Figure 2F,G).

### 2.3 Quantitative Comparison of Cellularity by THG Imaging and Fluorescence Imaging

**Figure 3:**
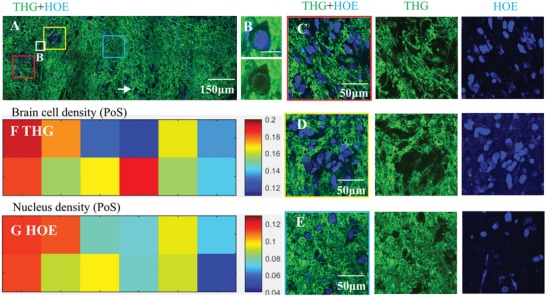
Validation of cell density present in THG images by HOE fluorescence imaging. HOE dye (staining DNA) was used to highlight all cell nuclei, and three‐photon fluorescence images (blue) with nuclei highlighted were recorded simultaneously with THG images (green). A) A 2 × 6 mosaic image of a glioma tissue sample, taken at a tissue depth of around 10 µm. The cell density roughly decreases from left to right (red, yellow, and blue squares). B) An example where a cell nucleus was colocated by THG and HOE staining. Scale bar: 20 µm. C–E) Magnified high cellularity and low cellularity areas marked on image (A). F,G) The densities of brain cells and nuclei (indicated by PoS) in the THG and HOE fluorescence images correlated well (correlation coefficient 0.71, *p*‐value 0.01), except the location (arrow) where high cell density was observed in THG while relatively lower nucleus density was observed in fluorescence. (The exclusion of this location resulted in correlation coefficient 0.85, *p*‐value 9.5e−04.) 3D scans (Figure S2, Supporting Information) collected at three locations (red square, yellow square, and arrow) suggested that the uneven uptake of fluorescence dyes occurred in this tissue, which may well explain the mismatch (arrow) between THG and HOE images in this case.

Our next step was to validate the cell density in glioma tissue displayed by THG imaging in a quantitative manner. One‐to‐one correlation of the cell density revealed by THG imaging with the H&E standard in the same tissue area is difficult, because the fixation process leads to shrinkage of the tissue and changes in cell morphology (e.g., compare cell size in Figure 1 in THG and in H&E images). Therefore, we compared THG one‐to‐one with three‐photon excited fluorescence imaging to demonstrate that the tissue cellularity observed in THG reflects the real cellularity of the tissue. Glioma tissue samples from two patients were stained with HOE to highlight cell nuclei. There was a good correlation between brain cells appearing as dark holes in THG images of these glioma samples and the cell nuclei highlighted by HOE (one example shown in **Figure** **3**A). Occasionally, the cell nuclei were also clearly delineated in THG images (Figure 3B). Both densely infiltrated tumor areas (Figure 3C,D) and areas with normal cell density (Figure 3E) were observed in these samples, with a decreasing gradient of invasive tumor cells from clear‐cut tumor areas toward normal tissue. Quantification of dark holes in the THG images and cell nuclei in the fluorescence images indicated that the density of brain cells quantified in THG images correlates well (correlation coefficient 0.71, *p*‐value 0.01 for the shown example) with the nucleus density quantified in fluorescence images (Figure 3F,G), except for one area of high cell density where a mismatch was found (Figure 3A, arrow). In this latter area, the number of identified cells in the fluorescence image was lower than in the THG image. This mismatch of cell density can at least partly be explained by the fact that in 3D scans of this area (and other tumor areas) (Figure S2, Supporting Information) uneven uptake of fluorescence dyes occurred.

### 2.4 Glioma Infiltration Quantitatively Indicated by THG Cellularity

After demonstrating that THG reliably reflects the true cellularity of brain tissue, we further explored the feasibility of detecting glioma infiltration quantitatively by using THG cellularity. Two density thresholds (Figure 2D,E) were generated from the statistical analysis of the first group of patients to discriminate THG images of normal brain from low‐grade gliomas and high‐grade gliomas, and thus to reveal glioma infiltration indicated by THG cellularity. We use a density threshold of brain cells at 0.17 to differentiate THG images of normal brain from histologically low‐grade glioma tissue (Figure 2D), and a density threshold of nuclei at 0.03 to distinguish THG images of normal brain from those of histologically high‐grade glioma tissue (Figure 2E). Mosaic THG images with the presence of both normal brain and glioma tissue obtained from patients 1–10 but excluded from the statistical analysis were used to generate the two density thresholds, by optimizing the best classification of each image tile of the mosaic images. An individual THG image was then classified as tumor if the density of brain cells was higher than 0.17 or the nucleus density was higher than 0.03, and otherwise, the image was designated as normal.

**Figure 4:**
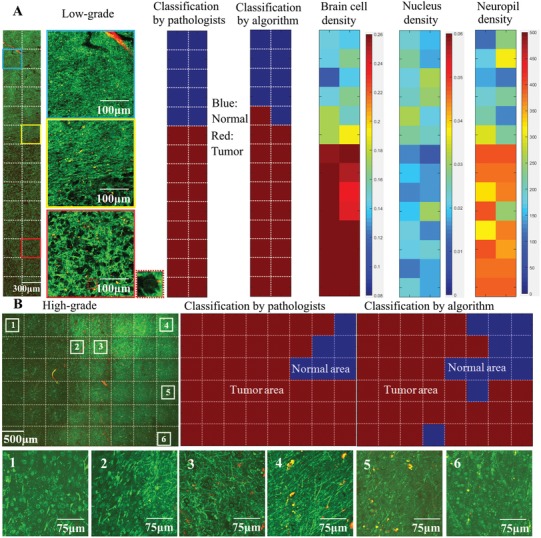
Glioma infiltration indicated by quantitative THG‐based assessment of cellularity. A) A 15 × 2 mosaic image (total image acquisition time: 3.5 min) of a histologically low‐grade diffuse glioma, with tumor cells appearing as dark holes with visible nuclei (red dash square).The cell density increases from normal brain (blue square) to tumor core (red square). The tumor boundary (yellow square) was correctly identified by quantitative THG analysis with high concordance with the classification by neuropathologists. B) A 6 × 8 mosaic image of an infiltrative high‐grade glioma, with total image acquisition time of 6 min, showing mild vascular proliferation and hypercellularity. The proliferation of tumor cells induced obvious intensity difference between the normal (square 4) and tumor areas (squares 1 and 6), because of the absence of lipid‐rich myelinated neuropil in tumor areas. An obvious boundary of the tumor core was observed between column 3 and column 4 (square 2), with some tumor cells invading into the surrounding brain tissue (square 3). A relative smooth transition from normal areas (square 4) to tumor areas (squares 5 and 6) was vertically observed on the right. Both the tumor regions on the left and bottom were correctly identified by quantitative THG, with high concordance with the consensus assessment by neuropathologists. Most disagreement between automated classification and neuropathologists occurred in the transition areas. Note that THG images in this case had a field of view of 500 × 500 µm^2^, and each of them was split into four equal parts and each part was classified by the algorithm. The computed features of the four parts were averaged to represent the features for the 500 × 500 µm^2^ image.

An independent THG dataset containing 839 THG images obtained from patients 11–23 was used to test the accuracy of classifying THG images with the two thresholds. This dataset consists of representative 2D THG images acquired from different tissue samples, as well as mosaic images of tissue samples in which normal brain, tumor infiltrated areas, and tumor core are observed (**Figure** **4**). Using the preoperative diagnosis and/or the corresponding H&E images as a definitive proof of the tissue state, a trained observer labeled each of the 839 THG images as “normal” or “tumor” as ground truth. The automatic classification algorithm with the two thresholds then classified each THG image as normal or tumor, which resulted in differentiation of tumor infiltrated areas from normal brain with sensitivity of 96.6% and specificity of 95.5%. The quantification and classification was applied on each square of the mosaic images, and quantitative THG was able to differentiate normal brain from tumor core and tumor infiltrated areas in both low‐grade (Figure 4A) and high‐grade tissue (Figure 4B). In both cases, the proliferation of tumor cells induced obvious intensity differences between the normal and tumor areas. A clear disruption of extracellular matrix was observed in the middle columns (Figure 4B, square 2) of the high‐grade case, with a few tumor cells invading further into the adjacent brain parenchyma (Figure 4B, square 3). This disruption may be due to the invasive tumor cells that dissolute the extracellular matrix during tumor cell invasion. Based on quantification of the cell density, quantitative THG was able to differentiate the tumor core (Figure 4B, squares 1 and 6) and the tumor infiltrated areas (Figure 4B, squares 3 and 5) from the normal areas (Figure 4B, square 4).

We further tested how the glioma infiltration detected by quantitative THG compared to that recognized by trained neuropathologists. A survey of 100 THG images (including individual representative images of samples and mosaic images having both normal and tumor areas) from patients 11–23, accompanied by six “training” examples of THG images of normal brain and gliomas, was presented to neuropathologists with ample expertise in the histological diagnosis of gliomas. They were asked to classify each THG image as normal or tumor. The classification by the algorithm and that by pathologists were double‐blind for each side. Comparison showed a nearly perfect agreement (93%) between the automated classification and the consensus of neuropathologists, and on average, the agreement between either two pathologists was 84% (Figure 4; Table S3, Supporting Information). This survey not only demonstrates that THG images can be efficiently interpreted by neuropathologists, but also inversely reflects the performance of the automated classification by the two thresholds.

### 2.5 Presence/Absence of Tumor in THG Images Correlates with Clinical Imaging

**Figure 5:**
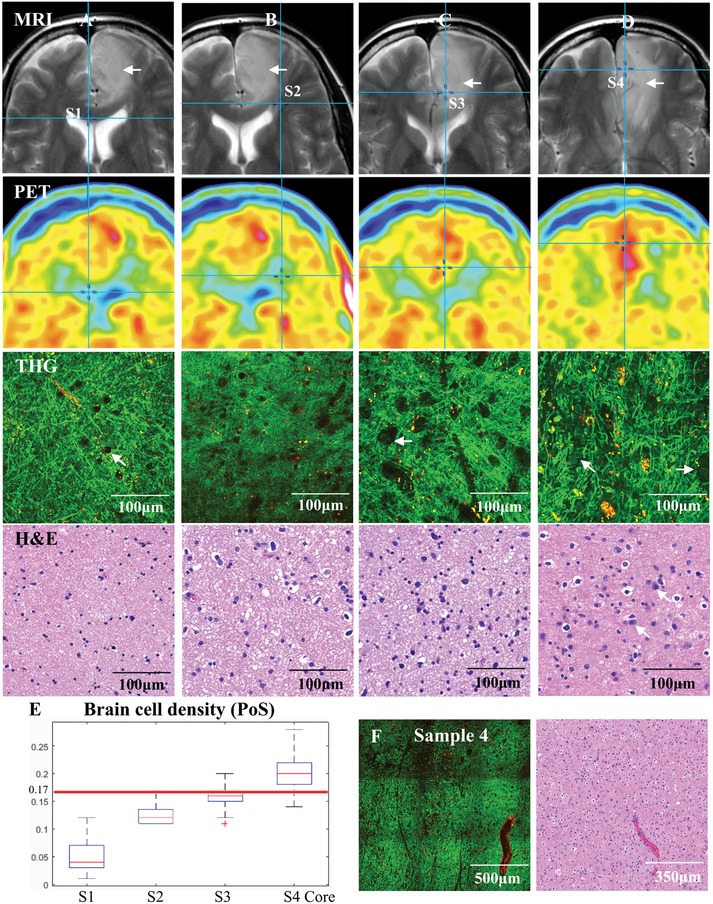
THG imaging of a grade II astrocytoma (IDH‐mutant) colocated on preoperative T2‐weighted MRI. A–D) Corresponding MRI, PET, THG, and H&E images of the four samples from patient 19. The coordinates of the four samples are indicated by a cross in the MRI. The contrast‐enhancing part of MRI (arrow) shows the tumor location. Sample 1 (S1) was removed from peritumoral (supposedly normal) brain, sample 2 (S2) was removed from tumor periphery, and samples 3 and 4 (S3, S4) were removed from the tumor. Normal cell density was observed in sample 1 in which brain cells appeared as dark holes (THG, arrow). An area with slightly increased cellularity was identified in sample 2. Mild hypercellularity was observed in sample 3. Moderately infiltrated areas and histopathological hallmarks of low‐grade astrocytoma, e.g., atypical‐appearing astrocytes with nuclear pleomorphism and enlargement (THG, arrow) were indentified in sample 4. The PET images suggest that cell metabolism increases from sample 1 to sample 4. This is fully consistent with the finding of THG that sample 1 and most parts of 2 are normal, and that tumor sample 4 is more densely infiltrated by tumor cells than tumor sample 3. E) The brain cell density quantified from THG images of S1–S4, demonstrating that the cell density increased from sample 1 to sample 4. F) Large blood vessels were used to correlate THG and H&E images, showing hypercellularity of sample 4.

We lastly correlated THG imaging with clinical modalities to illustrate the potential clinical application of THG, e.g., instant tissue pathology during surgery. Four tissue samples were removed from a patient with low‐grade IDH‐mutant astrocytoma at different locations varying from peritumoral (supposedly normal) brain, tumor periphery, and tumor core (**Figure** **5**). The coordinates of the four samples were recorded during operation in reference to presurgical MRI scans. In sample 1, obtained at a relatively large distance from the tumor as delineated in preoperative T2 MRI, the THG images showed typical features of white matter with normal cell density and densely structured neuropil (Figure 5A,E; Figure S3A, Supporting Information). In sample 2, obtained close to the tumor border as seen on T2 MRI, THG imaging found predominantly normal brain and a small area with slightly increased cellularity (Figure 5B,E), highly indicative for invasion by a limited number of glioma cells (Figure S3B–D). In sample 3, THG imaging found normal white matter in ≈70% of the sample area and tumor areas with mildly increased cellularity in ≈30% (Figure 5C,E; Figure S3E, Supporting Information). The tumor cells appeared as dark holes with increased size, and occasionally their cell nuclei were visible (Figure 5C, arrow; Figure S2F, Supporting Information). In sample 4, obtained from within the tumor, a moderate to strong increase of (tumor) cells was seen with THG imaging (Figure 5D,E), causing axonal disruption and small “cavities” filled with tumor cells (Figure S3G, Supporting Information). The tumor cells appeared as dark holes with atypical‐appearing and enlarged nuclei.

After THG imaging of these samples, the tissue state was also analyzed by performing microscopic evaluation of H&E stained histological slides. Large blood vessels were used as “landmarks” for identification of the same tissue area in the THG and H&E images (Figure 5F). The apparent diffusion coefficient (ADC) of the four samples, recorded from diffusion MRI,^40^ suggested that the cell density of sample 3 (0.0015400 mm^2^ s^−1^) and sample 4 (0.0013200 mm^2^ s^−1^) was on an average ≈40% higher than sample 1 (0.0012290 mm^2^ s^−1^) and sample 2 (0.0008282 mm^2^ s^−1^), which quantitatively agreed with THG imaging. 18F‐fluoro‐ethyl‐tyrosine (^18^F‐FET) positron emission tomography (PET) data suggested that cell metabolism increased from sample 1 to sample 4.^41^, ^42^ This supported the THG interpretation that sample 1 and most parts of sample 2 were normal, and sample 4 was more densely infiltrated by tumor cells than sample 3. These results demonstrate that the overall appearance of THG images is in agreement with the diffusion weighted MRI and ^18^F‐FET PET imaging. However, the subcellular resolution of THG shows heterogeneity of the tissue samples with areas of different cell densities. If used intraoperatively next to MRI, THG imaging thus has great potential for enabling more refined removal of areas with abnormal cell density and morphology.

## 3 Discusssion

Diffuse gliomas are highly invasive and they invade beyond the visible MRI borders.^43^ Surgery serves as the first‐line treatment of diffuse gliomas. Safely maximizing the extent of resection while sparing normal brain remains a challenge. Residual tumor cells give rise to recurrence, while overly ambitious gross total resection incurs surgery‐related deficits which also reduces median survival of patients.^5^, ^44^, ^45^ In order to improve the surgical outcomes of patients with a diffuse glioma, several label‐free optical techniques have been proposed to enable real‐time feedback of tissue state, but it is not yet clear which one is the most promising for clinical use.

This study demonstrates how THG microscopy can be used to quantitatively detect glioma infiltration in human brain tissue in ex vivo fresh, unprocessed surgical tissue samples. THG imaging of such samples from 23 patients revealed histology‐grade characteristics of normal brain and of WHO grades II–IV diffuse gliomas. Qualitative and quantitative comparison of THG with H&E and fluorescence data demonstrates the great potential of THG microscopy for detection of the presence of glioma infiltration by cellularity. The evaluation by the neuropathologists suggests that THG images can indeed be efficiently interpreted by neuropathologists.

In these kind of studies, the ultimate ground truth for tumor‐infiltrated versus noninfiltrated areas in a tissue sample is difficult to acquire. Preoperative imaging techniques such as MRI suffer from brain shift and brain surface deformations after craniotomy. One‐to‐one comparison of THG with H&E images is difficult because of the fixation and slicing process. Fluorescence imaging suffers from uneven staining that also makes it difficult to do one‐to‐one comparison between THG and fluorescence imaging in a large image scale. Here, we choose the strategy to colocalize and/or correlate THG imaging with all these imperfect ground truths and show the ability of THG microscopy to directly visualize the decreasing gradient of invasive tumor cells, spreading from the main tumor mass into normal tissue.

Correlating THG imaging with MRI, PET, and H&E analysis not only illustrates the potential of THG microscopy as an auxiliary tool for fast diagnosis of tissue to be excised, but also suggests THG as a potential tool for tumor preliminary typing during the biopsy procedure. We have shown the substantial morphological differences in THG images of different glioma types. THG can also reveal diagnostic features of extrinsic brain tumors, e.g., meningioma (Figure S4, Supporting Information). THG microscopy thus is promising for the selection of representative tumor tissue that can be used for more detailed and more accurate (molecular) diagnosis of brain tumors. Meanwhile, we are fully aware that substantially more patient data are needed to demonstrate the validity of tumor typing with THG.

Although other label‐free imaging techniques such as optical coherence tomography and Raman techniques have also been shown to be able to differentiate tumor infiltration,^17^, ^18^, ^19^, ^20^, ^21^, ^46^ THG has its own distinctive merits. THG provides a higher resolution and richer cellular morphology that is currently unfeasible for swept‐source OCT.^17^ Full‐field OCT has higher resolution than swept‐source OCT,^46^ but the resolution achieved is still not sufficient to reveal tissue morphology in a way comparable to THG. It has also been reported that full‐field OCT is not sensitive for detection of tumor infiltration.^46^ A challenging limitation of Raman spectroscopy is the lack of tools to help with histopathological interpretation based on subtle differences in the vibrational spectra of tumor tissue and normal tissue.^18^ The implementation of THG microscopy is less complicated and less costly than Raman microscopies because Raman microscopies require two laser sources to be overlapped spatially and temporally.^19^, ^20^, ^21^ More importantly, in the sense that THG microscopy directly visualizes rich cellular morphology of fresh tissue, THG could generate more reproducible data and thus is potentially more reliable than Raman microscopies, which visualize cellular morphology via the relative intensity of vibrational bands indicative of lipids and protein.

Currently, compact and transportable THG microscopes using low laser power and with high scanning rates that can be used for real‐time ex vivo tissue assessment in the operation room are appearing in the market. Employing fiber lasers operating at lower (1–30 MHz) repetition rates instead of the optical parametric oscillators that currently are used for most THG imaging, not only THG microscopes can be minimized to a table size of ≈1 × 1 × 1 m^3^, but also the laser power needed for efficient generation of THG/SHG signals can be reduced to 14 mW.^47^ Importantly, we have reported previously THG endomicroscopic imaging of brain tumor tissue with low laser power (50 mW), as a step toward in situ THG microendoscopy of brain tumors.^28^ Moreover, with optimized laser scanning strategies and low laser power, frame rates of >0.5 Hz have been achieved for an area of field of view ≈300 × 300 µm^2^ and with ≈600 × 600 pixels.^47^ Further optimization is expected to lead to frame rates of around 10 Hz, enabling handheld in situ imaging with a microendoscopic device.

In summary, we demonstrate the power of ex vivo quantitative THG microscopy in detecting and quantifying diffuse glioma infiltration based on the assessment of tissue cellularity. Correlation with multiple clinical imaging modalities demonstrates the feasibility and efficiency of using THG microscopy as a potential imaging tool next to MRI, either ex vivo or in situ. Thereby, quantitative THG microscopy has the potential to improve the accuracy of brain tumor surgery.

## 4 Experimental Section

*Study Design*: The objective of this study was to evaluate the potential of THG microscopy to quantitatively distinguish glioma infiltration from normal brain tissue. 56 (10 normal, 46 glioma) tissue samples were removed from 23 patients with epilepsy (*n* = 7) or grades II–IV diffuse gliomas (*n* = 16). Both male and female adult patients with ages from 20 to 70 were included in the study to reduce the dependence of data on gender and age. All patients gave a written informed consent for tissue biopsy collection and signed a declaration permitting the use of their biopsy specimens in scientific research. All procedures on human tissue were performed with the approval of the Medical Ethical Committee of the VU University Medical Center (VUmc) and in accordance with Dutch license procedures and the declaration of Helsinki. The preoperative diagnosis was based on MRI imaging, the diagnosis of normal brain tissue or of glioma was based on histological analysis, and in case of glioma with additional molecular testing. The tissue sample size was ≈1 cm × 1 cm × 0.5 cm and was chosen based on the design of previous studies comparing THG and H&E imaging modalities. THG imaging was performed at the laboratory of Vrije Universiteit Amsterdam located within 500 m from VUmc. After THG imaging, the tissue samples were fixated in 4% formaldehyde and returned to the Pathology department of VUmc for standard H&E analysis. A trained neuropathologist (P.W.) histologically classified the samples as normal or tumor.

To quantify THG images, an automatic quantification workflow was implemented in Visual Studio C++2010. The automatic classification results were compared with the classification from three neuropathologists (A.R., P.W., and W.V.), through a double‐blinded survey. The survey consisted of a “training” dataset with six examples to briefly introduce the cell morphology present in THG images of normal brain and gliomas, and a “testing” dataset with 100 THG images to be classified as normal or tumor.

*THG Microscopy*: THG is a nonlinear optical process that depends on the third‐order susceptibility χ^(3)^ of the tissue and certain phase‐matching conditions.^23^, ^48^ The lipid‐rich components of brain tissue are the major source of contrast for THG.^24^, ^26^ SHG signals, arising from noncentrosymmetric structures, provide information complementary to THG, e.g., blood vessels. The THG microscope used for imaging human brain samples has been previously described and is shown in Figure S5 in the Supporting Information.^26^ The laser source was an optical parametric oscillator (OPO) pumped at 810 nm by a Ti‐sapphire oscillator, generating 200 fs pulses at 1200 nm and repetition rate of 80 MHz. The beam from OPO was delivered into a commercial two‐photon laser‐scanning microscope and focused on the sample using a 25×/1.10 water‐dipping objective. The long laser wavelength (1200 nm) used to generate the THG/SHG signals resulted in deep penetration depth and ensured a low risk of damaging the imaged tissue, while the short wavelengths resulting from the THG/SHG processes (400/600 nm) enabled efficient back‐scatter detection. The signals from the sample were filtered from the 1200 nm fundamental photons by a dichroic mirror, split into SHG and THG channels by a dichroic mirror, and passed through narrow‐band interference filters for SHG and THG detection by high‐sensitivity GaAsP photomultipliers. In case of imaging with THG and HOE three‐photon fluorescence, the narrow‐band filter for SHG was replaced by a broad‐band filter (430–570 nm, Chroma, HQ500/140 M‐2P) for collection of HOE signals.

An average laser power of 100 mW was used on the tissue sample to generate THG/SHG signals. The image acquisition time for each pair of THG and SHG images, having a field of view ≈300 × 300 µm^2^ with ≈1000 × 1000 pixels, was ≈8 s. The mosaic imaging of the sample was performed by transverse (*xy*) scanning of the motorized translation stage. Images were recorded and stored using TriMScope I software (“Imspector Pro”), and the adjacent THG/SHG images had 20% overlap. According to this fixed overlapping rate, the stitching algorithm provided by ImageJ software (version 1.49m, NIH, USA) was used to stitch adjacent THG/SHG image tiles into mosaic images.

*Tissue Preparation*: After tissue resection, brain tissue samples were placed within 30 s in ice‐cold artificial cerebrospinal fluid (ACSF) at 4 °C, and transported to the laboratory within 15 min. There, the samples were cut with a surgical scalpel to generate flat surfaces, rinsed with ACSF to remove blood contamination, embedded in agar blocks, and flattened with a 0.17 mm thick glass cover slip for THG imaging. For the fluorescence staining experiments, three drops of Hoechst‐33342 dye were put into 1 mL of ACSF and the tissue slices were incubated for 15–20 min to label nuclear DNA of brain cells.

*Quantification Workflow and Statistical Analysis*: Here, an integrated workflow (Figure 2A) to quantify brain cells, cell nuclei, and neuropil present in THG images of normal brain and glioma tissue is proposed. THG images were first preprocessed by histogram truncation and local histogram equalization to enhance the global and local image contrast.^49^ The enhanced images were denoised by salient edge‐enhancing anisotropic diffusion,^38^ which was able to remove the noise while keeping the edges sharp. 3‐phase active contour weighted by prior extreme was applied for segmenting dark holes and bright objects present in THG images.^38^ This segmentation algorithm was able to detect all dark holes and most of bright objects, but some bright nuclei were not detected by this segmentation algorithm because the nuclear contrast was sometimes too weak. The background was then further processed by a slightly modified version of circular Hough transform to extract those round or elliptical nuclei that were not detected by the 3‐phase segmentation algorithm.^50^ The implementation of circular Hough transform contained a voting process that mapped image edge points into an accumulation matrix. Peaks in the accumulation corresponded to the center of the circles. Only the edge pixels voting was allowed along the gradient direction. The HOE fluorescence images were quantified similar to the detection of bright objects in THG images.

To quantify the dark holes, hole filling was applied to complete the insides of dark holes and an object splitting algorithm was applied to separate slightly touching objects.^33^ Dark holes with size less than 1000 pixels (≈6 µm × 6 µm) were ignored and the remaining objects were considered as brain cells or cell cytoplasm. For the quantification of neuropil and cell nuclei, all the bright objects with sizes less than 500 pixels were ignored. The remaining bright objects were sorted by sphericity/compactness.^39^ Bright objects with sphericity <0.1 were considered as neuropil. Object splitting algorithm was applied to the remaining bright objects to separate the slightly touching nuclei.^33^ The bright objects of sphericity >0.5 and size <10 000 pixels were combined with the nuclei detected in the background, as the nuclei detected in the whole image. The brain cells/cytoplasm appearing as dark holes and bright nuclei were combined as detections of brain cells (Figure 2C). The density of a feature was represented by the percentage of space taken by that feature.^38^ For example, PoS of nuclei is the total number of pixels of the detected nuclei divided by the image size in pixels. PoS is a combined parameter of object number and object size. It is an adequate representation of each pathological feature when the number of feature is extremely high and the object clumps are difficult/impossible to separate. The computational time needed for processing a THG image was ≈1 min using a single CPU core (3.40 GHz Intel(R) Core(TM) i7‐2600) and ≈10 s using 8 CPU cores.

The statistical analysis of the densities of brain cells, nuclei, and neuropil (Figure 4D–G) were performed on 800 representative THG images of normal brain (*n* = 140), low‐grade (*n* = 414), and high‐grade (*n* = 246) glioma tissue samples obtained from patients 1–10. Mosaic THG images with the presence of both normal brain and glioma tissue obtained from patients 1–10 but excluded from the statistical analysis were used to generate the density thresholds to differentiate normal brain from glioma areas. An independent THG dataset containing 839 THG images obtained from patients 11–23 was used to test the accuracy of classifying THG images with the two thresholds, in terms of sensitivity and specificity.

## Conflict of Interest

The authors declare no conflict of interest.

## Supporting information

Supplementary
