# Supplementary material for: Quantitative Third Harmonic Generation Microscopy for Assessment of Glioma in Human Brain Tissue
Source: Adv Sci (Weinh). 2019 Apr 5;6(11):1900163. doi: 10.1002/advs.201900163 (PMC6548968; doi:10.1002/advs.201900163)
Supplement: Supplementary file 1 — Supplementary [file ADVS-6-1900163-s001.pdf]

Copyright WILEY-VCH Verlag GmbH & Co. KGaA, 69469 Weinheim, Germany, 2019.

ADVANCED SCIENCE
Open Access

Supporting Information

for *Adv. Sci.,* DOI: 10.1002/advs.201900163

Quantitative Third Harmonic Generation Microscopy for Assessment of Glioma in Human Brain Tissue

*Zhiqing Zhang, Jan C.de Munck, Niels Verburg, Annemieke J. Rozemuller, Willem Vreuls, Pinar Cakmak, Laura M. G.van Huizen, Sander Idema, Eleonora Aronica, Philip C.de Witt Hamer, Pieter Wesseling, and Marie Louise Groot**

Supporting Information

Quantitative third harmonic generation microscopy for assessment of glioma in human brain tissue

*Zhiqing Zhang, Jan C. de Munck, Niels Verburg, Annemieke J. Rozemuller, Willem Vreuls, Pinar Cakmak, Laura M. G. van Huizen, Sander Idema, Eleonora Aronica, Philip C. de Witt Hamer, Pieter Wesseling, Marie Louise Groot**

Table S1. The diagnoses of the patients. The preoperative diagnosis was based on MRI imaging, the diagnosis of normal brain tissue or of glioma was based on histological analysis, and in case of glioma with additional molecular testing.

GM: gray matter; WM: white matter;

Patient ID	Diagnosis	Number of biopsies (total 56)
1	Structurally normal neocortex/GM and subcortical WM	1
2	Structurally normal neocortex/GM and subcortical WM	2
3	Left-frontal diffuse astrocytoma II	1
4	Left fronto-parietal oligo-astrocytoma II	4
5	Right insular oligodendroglioma II	3
6	Left parietal oligodendroglioma II	3
7	Right insular oligodendroglioma II	4
8	Left-frontal low-grade glioma	3
9	Glioblastoma	3
10	Glioblastoma	2
11	Structurally normal neocortex/GM and subcortical WM	1
12	Structurally normal neocortex/GM and subcortical WM	1
13	Structurally normal neocortex/GM and subcortical WM	1
14	Structurally normal neocortex/GM and subcortical WM	2
15	Anaplastic Oligodendroglioma III	4
16	Glioblastoma (IDH1-wildtype)	2
17	Astrocytoma II (IDH1-mutant)	2
18	Recurrent Glioblastoma	2
19	Astrocytoma II (IDH1-mutant without 1p19q codeletion)	4
20	Glioblastoma	4
21	Low-grade glioma	2
22	Low-grade glioma	3
23	Structurally normal neocortex/GM and subcortical WM	2

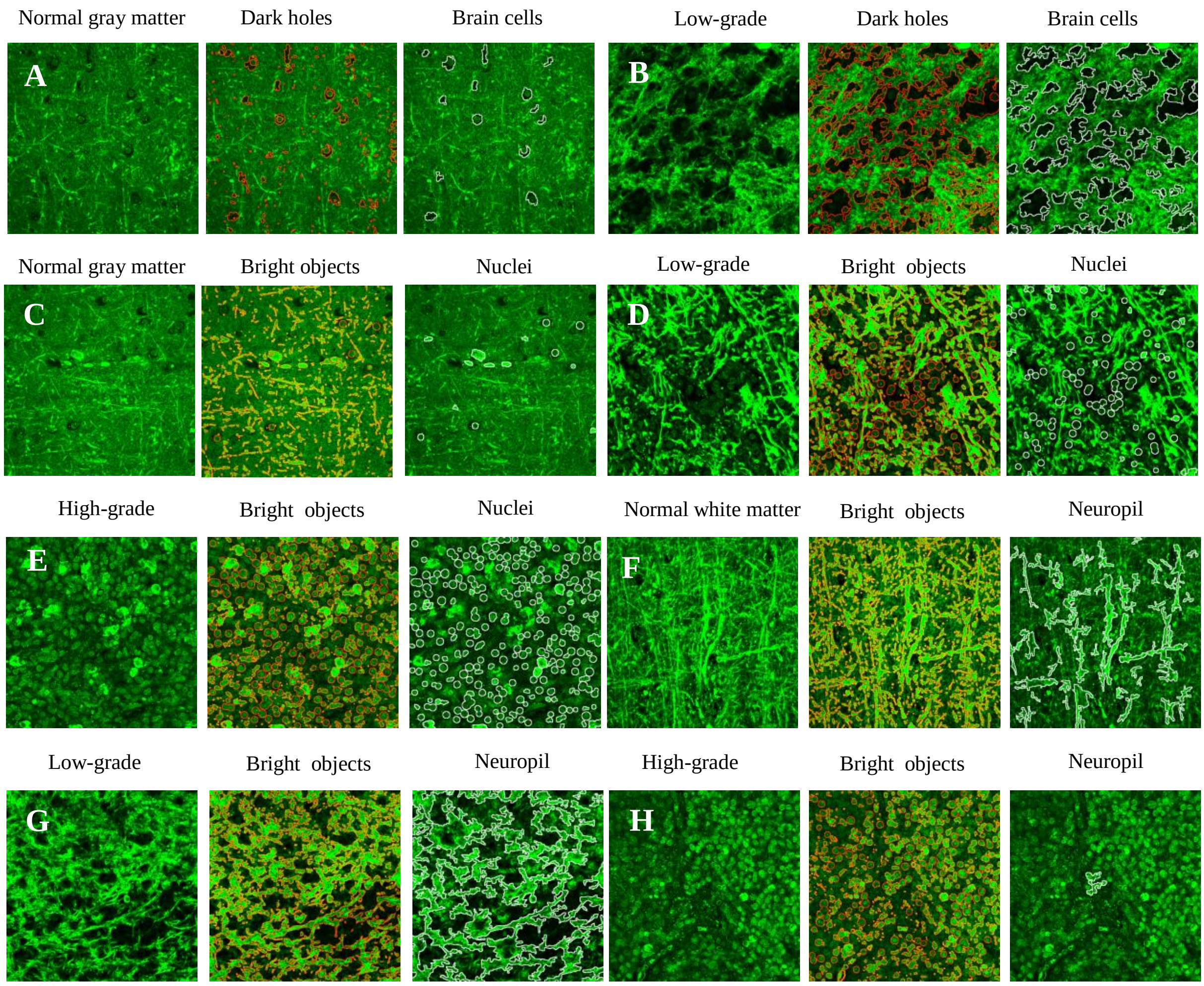

Figure S1. Illustration of the validation of THG image segmentation. Each THG image was segmented into dark holes and bright objects. After segmentation, small dark holes were ignored, and the remaining dark holes were considered as brain cells/cytoplasm. Small bright objects were also ignored, and sphericity was used to separate the nuclei and fiber-like neuropil (see main text for applied thresholds). A software tool was developed to overlay the raw THG images with the dark holes, bright objects, brain cells/cytoplasm, nuclei and neuropil detected. Each detected object was checked if it represents a THG object and if the THG object had been correctly segmented. **(A-B)** Segmentation of brain cells/cytoplasm in normal brain and low-grade glioma tissue samples. **(C-E)** Segmentation of cell nuclei in normal brain, low-grade glioma and high-grade glioma tissue samples. **(F-H)** Segmentation of neuropil in normal brain, low-grade glioma and high-grade glioma tissue samples.

Table S2. Detailed validation of THG image segmentation. 10 representative images from normal brain, 10 from low-grade glioma and 10 from high-grade glioma tissue samples were used for validation. The dark holes (of size ≥ 500 pixels) and bright objects (of size ≥ 200 pixels) detected were overlaid with the raw THG images and each detected object was checked to make sure the candidates of brain cells, nuclei and neuropil being detected correctly. Dark holes larger than 1000 pixels were considered as brain cells/cytoplasm, and each brain cell/cytoplasm was checked if it represented a real brain cell or a cell clump. Sphericity was used to select nuclei and fiber-like neuropil from bright objects detected (of size ≥ 500 pixels). The number of resulting nuclei was compared with manual nucleus counting. The density of resulting neuropil was measured by their perimeter length. The total length was re-normalized to a 5-point scale based on our full THG image library and compared to manual rating on a 5-point scale ([0,50) , [50,100), [100,200), [200,300), [300,∞)). In total, the segmentation algorithm was able to accurately detect all the dark holes (2nd main column, Dark holes) and bright objects (3rd main column, Bright objects) observed within THG images. Most (2116/2147=98.6%) dark holes above the size threshold appearing in THG images of normal brain, low-grade and high-grade gliomas represented brain cells or cell cytoplasm (4th main column, Dark brain cells/Cytoplasm). The feature separation step was able to separate most of neuropil fibers which agreed well with manual rating (6th main column, Neuropil). The nucleus density is the key difference between THG images of normal brain and high-grade gliomas. The number of nuclei detected from THG images of high-grade gliomas was on average 5% (at most 15%) lower than the manual nucleus counting (5th main column, Nuclei/Round objects, HG1-HG10), due to nucleus clumps that were misclassified as fiber structures and the low contrast of some nuclei in corners (Figure S1 H). Because some fragments of neuropil with round shape were recognized as cell nuclei by the algorithm, the nucleus density detected from THG images of normal brain was on average18% higher than the manual nucleus counting (5th main column, Nuclei/Round objects, N1-N10). However, this resulted in an overestimation of only 7 cell nuclei per image maximally.

N: normal brain; LG: low-grade; HG: high-grade; TP: true positive.

Image Index	Dark holes		Bright objects		Dark brain cells/Cytoplasm		Nuclei/Round objects		Neuropil	
	Total	TP	Total	TP	Total	TP	Manual	Auto	Manual	Auto
N1	25	25	178	178	14	13	14	18	Scale 1	5 (Scale 1)
N2	9	9	127	127	2	2	9	9	Scale 1	21 (scale 1)
N3	147	147	220	220	66	66	25	32	Scale 4	175 (Scale 3)
N4	21	21	176	176	13	11	11	13	Scale 1	0 (Scale 1)
N5	150	150	209	209	84	84	32	38	Scale 4	249 (Scale 4)
N6	19	19	111	111	3	3	2	2	Scale 2	56 (Scale 2)
N7	160	160	201	201	90	90	17	20	Scale 5	290 (Scale 4)
N8	146	146	181	181	81	81	18	23	Scale 5	271 (scale 4)
N9	63	63	116	116	6	6	5	6	Scale 2	72 (Scale 2)
N10	143	143	234	234	77	77	21	28	Scale 4	191 (Scale 3)

LG1	120	120	192	192	118	118	154	146	Scale 4	222 (Scale 4)
LG2	92	92	66	66	58	58	20	19	Scale 5	362 (Scale 5)
LG3	123	123	126	126	94	94	21	22	Scale 4	275 (Scale 4)
LG4	126	126	157	157	115	115	128	122	Scale 4	290 (Scale 4)
LG5	89	89	53	53	68	67	12	15	Scale 5	351 (Scale 5)
LG6	140	140	146	146	100	94	20	23	Scale 4	285 (Scale 4)
LG7	94	94	67	67	74	74	18	22	Scale 5	355 (Scale 5)
LG8	116	116	177	177	112	112	145	134	Scale 4	239 (Scale 4)
LG9	152	152	131	131	101	93	24	27	Scale 4	232 (Scale 4)
LG10	119	119	168	168	120	120	136	128	Scale 5	310 (Scale 5)
HG1	111	111	278	278	77	73	180	169	Scale 1	9 (Scale 1)
HG2	95	95	175	175	78	78	86	91	Scale 2	48 (Scale 2)
HG3	100	100	266	266	70	64	169	148	Scale 1	0 (Scale 1)
HG4	104	104	257	257	88	88	171	148	Scale 1	0 (Scale 1)
HG5	88	88	160	160	64	64	72	81	Scale 2	62 (Scale 2)
HG6	105	105	270	270	78	76	189	176	Scale 1	21 (Scale 1)
HG7	89	89	153	153	68	68	85	91	Scale 2	51 (Scale 2)
HG8	102	102	164	164	68	68	53	60	Scale 1	0 (Scale 1)
HG9	97	97	180	180	65	65	63	69	Scale 1	0 (Scale 1)
HG10	116	116	260	260	95	94	174	155	Scale 1	0 (Scale 1)
Total	3061	3061	5199	5199	2147	2116	2074	2035	--	--

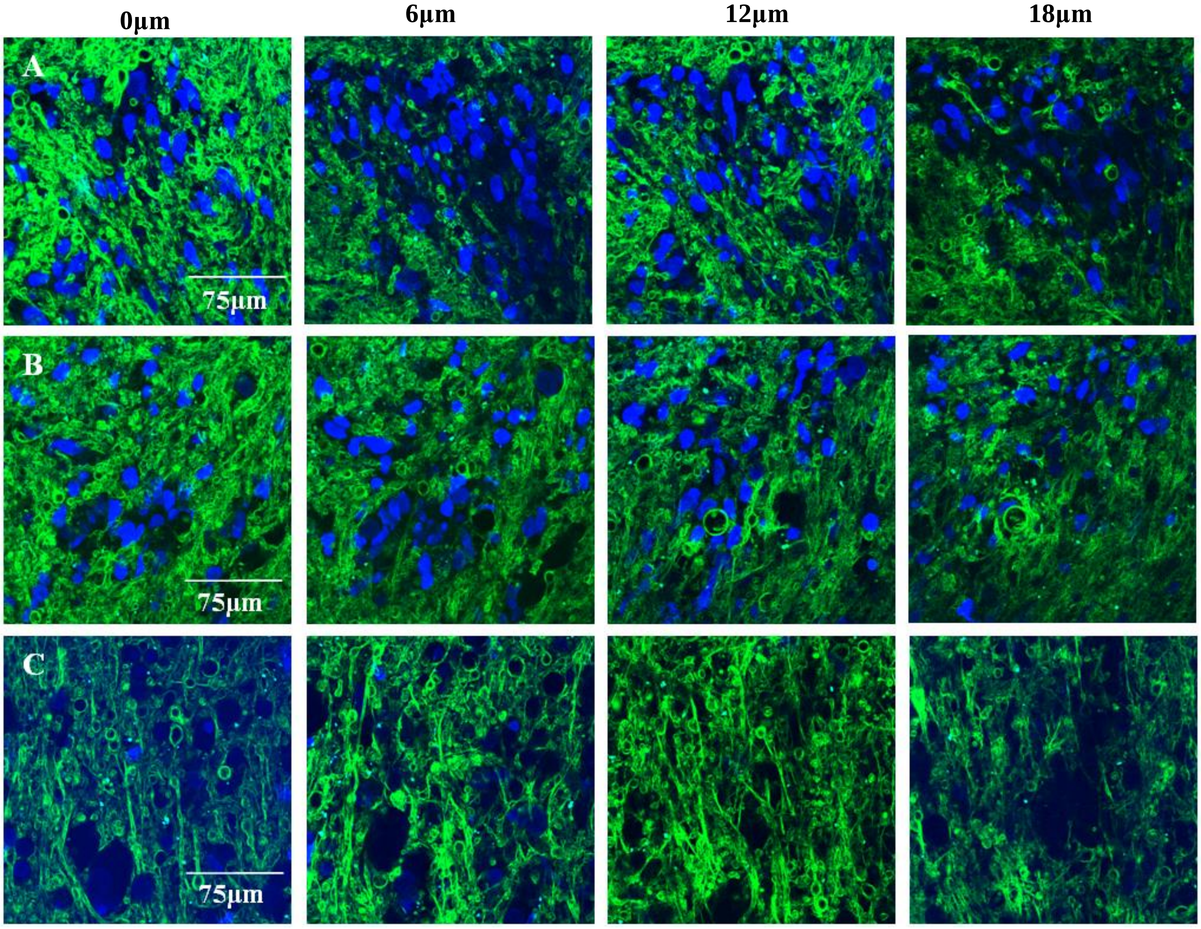

Figure S2. 3D THG images (green) of tumor areas overlaid with Hoechst fluorescence (blue) imaging. (A-B) 2D slices at depth 0, 6, 12, 18 µm of tumor areas showing that staining was complete in these areas and agreed with THG signals. **(C)** An example of tumor area where staining was not complete and mismatched with THG signals.

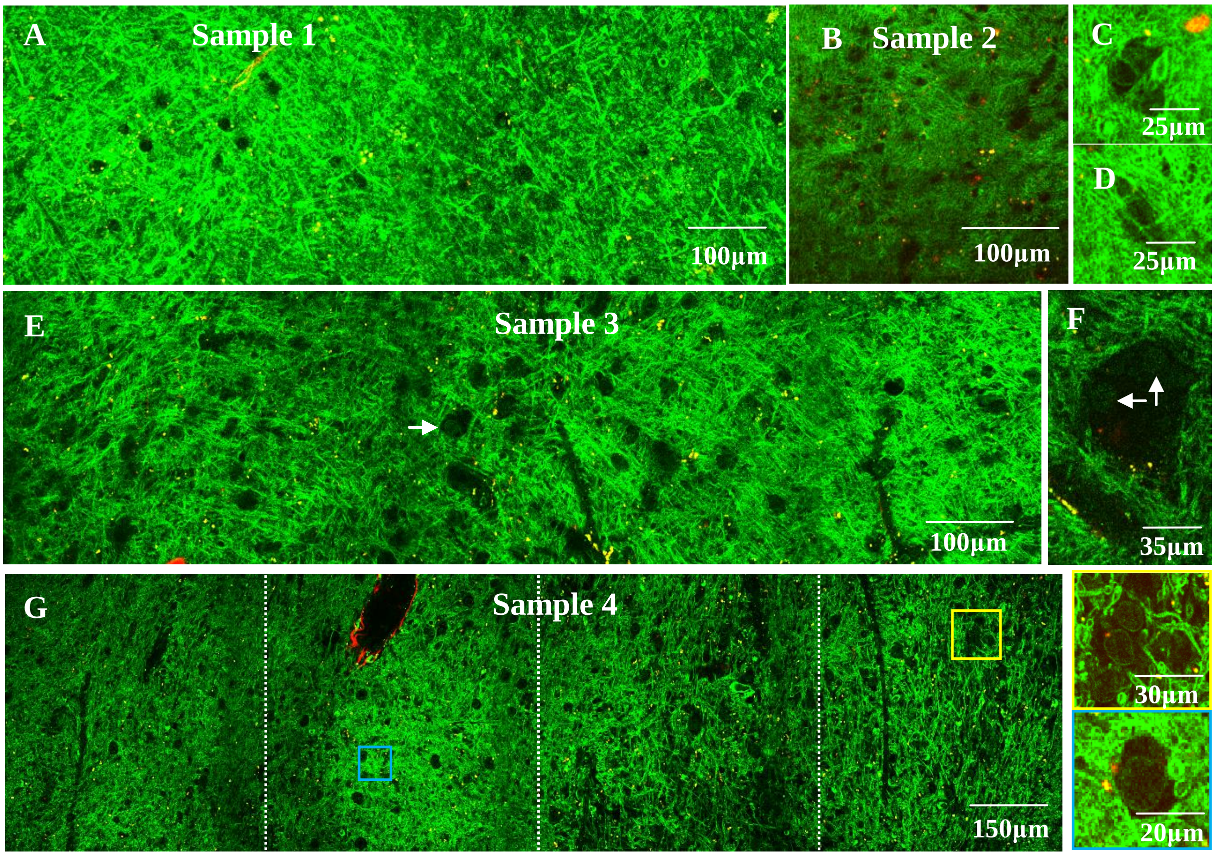

Figure S3. THG mosaic images of four samples from a grade II astrocytoma patient. Sample 1 was removed from peritumoral (supposedly normal) brain, sample 2 was removed from tumor periphery, and samples 3-4 were removed from the tumor site. **(A)** THG images of tissue sample 1 showing normal cell density. **(B-D)** THG imaging of tissue sample 2 showing slightly increased cellularity (B). Cell clumps (C-D) were observed that suspected to be a tumor area with a few invasive tumor cells. **(E-F)** THG imaging of tissue sample 3, showing areas with mild hypercellularity (E) and tumor cells with visible nuclei (arrow & F). **(G)** A mosaic image of sample 4, showing moderately (left) and densely (right) infiltrated areas, nuclear pleomorphism, nuclear enlargement (blue square) and small cavities filled with tumor cells (yellow square).

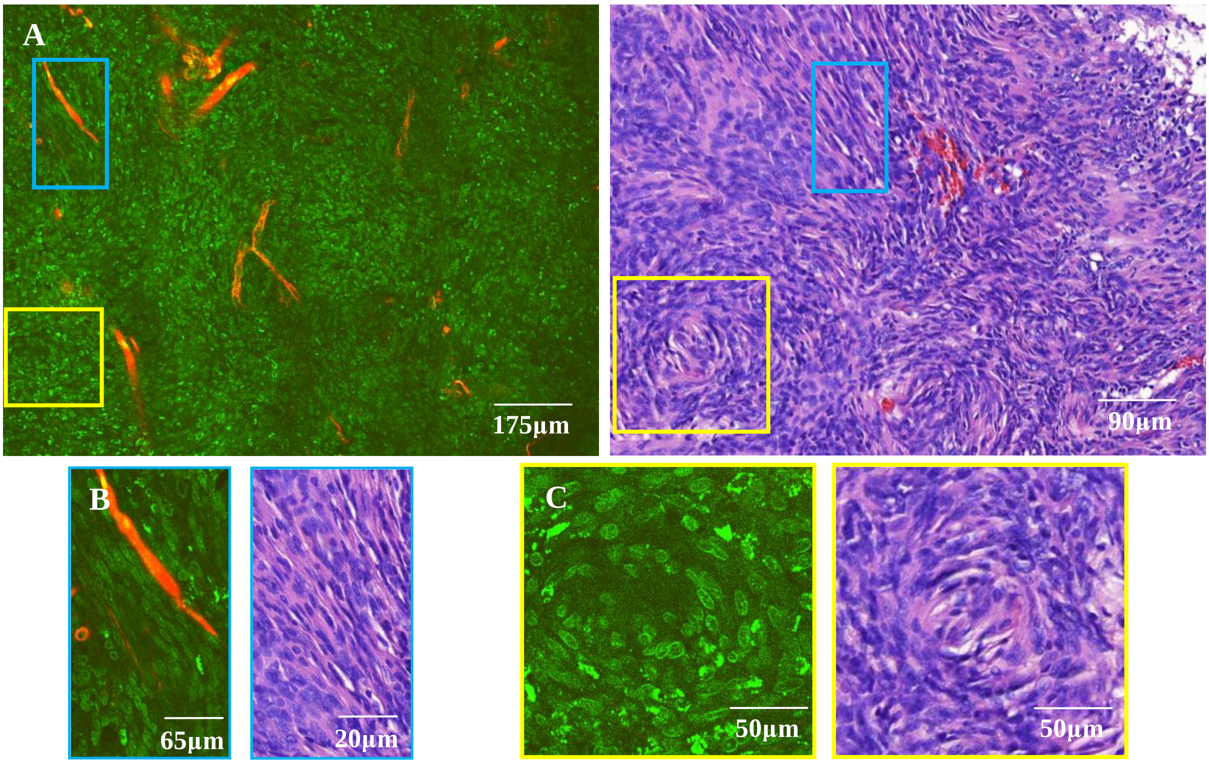

Figure S4. THG imaging of fresh, unprocessed meningioma tissue. Meningiomas are among the more common nonglial tumors of the central nervous system that are quite amenable to surgical excision. Here we compare THG images (green) and H&E images (purple) of the same sample to demonstrate that THG shows the same characteristics as the clinical standard. **(A)** Low-magnification appearance of the meningioma, showing spindled cells with elongated nuclei arranged in fascicle. **(B)** High magnification showing spindled cells arranged in fascicle. **(C)** Focal whirling of tumor cells, which is a characteristic feature of meningioma.

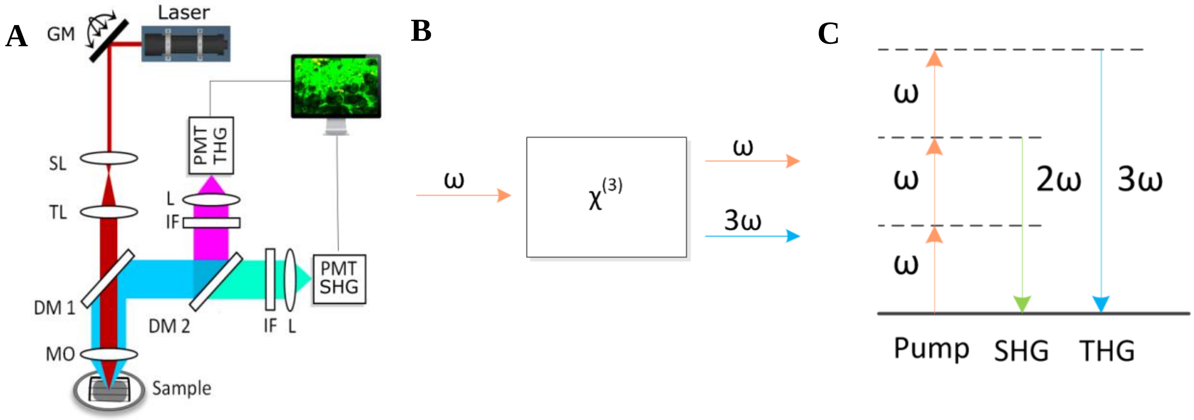

Figure S5. Third harmonic generation (THG) microscopy. (A) Schematic of THG microscopy setup. 1200nm laser beam was used to generate the THG and second harmonic generation (SHG) signals. THG/SHG signals were collected at 400/600 nm and were depicted in the images in green/red color. GM: Galvo mirror, SL: Scan lens, TL: Tube lens, DM: Dichroic mirror, MO: Microscope objective, IF: Interference filter, L: lens, and PMT: Photomultiplier tube. **(B)** Geometry of the THG process. **(C)** Energy level diagram of the THG process.

Table S3. The survey of comparison of pathologists and automated classification. 100 representative THG images were passed to 3 neuropathologists. Each neuropathologist independently classified the images as normal (1) or tumor (2). The automated classification was in 93% agreement with the consensus of neuropathologists. On average, the agreement between either two pathologists (A.R. VS P.W. 87%, A.R. VS W.V. 77%, P.W. VS W.V. 88%) was 84%.

Image No.	Neuropathologist A.R.	Neuropathologist P.W.	Neuropathologist W.V.	Consensus of neuropathologists	Automated classification
1	1	1	1	1	1
2	2	2	2	2	2
3	2	2	2	2	2
4	1	2	2	2	2
5	1	1	1	1	1
6	2	2	2	2	2
7	2	2	2	2	2
8	1	1	2	1	2
9	1	1	1	1	1
10	2	2	2	2	2
11	1	1	1	1	1
12	2	2	2	2	2
13	2	2	2	2	2
14	1	2	2	2	2
15	1	1	2	1	1
16	1	1	1	1	1
17	1	1	2	1	1
18	1	1	2	1	1
19	1	1	1	1	1
20	1	1	2	1	1
21	1	1	2	1	1
22	1	1	2	1	1
23	1	1	2	1	1
24	1	1	2	1	1
25	2	2	2	2	2
26	2	2	2	2	2
27	2	2	2	2	2
28	2	2	2	2	2
29	2	2	2	2	2
30	2	1	2	2	1
31	2	2	2	2	1

32	1	1	1	1	1
33	2	2	2	2	2
34	2	2	2	2	2
35	2	2	2	2	2
36	2	2	2	2	2
37	2	2	2	2	2
38	2	2	2	2	2
39	1	1	2	1	1
40	1	1	1	1	1
41	2	2	2	2	2
42	2	2	2	2	2
43	2	2	2	2	2
44	2	2	2	2	2
45	2	2	2	2	1
46	1	1	2	1	1
47	1	1	1	1	1
48	1	1	1	1	1
49	2	2	2	2	2
50	2	2	2	2	2
51	2	2	2	2	2
52	2	2	2	2	2
53	2	2	2	2	2
54	2	2	2	2	1
55	1	2	2	2	2
56	1	2	2	2	2
57	2	2	2	2	2
58	2	2	2	2	2
59	2	2	2	2	2
60	2	2	2	2	2
61	2	2	2	2	2
62	2	2	2	2	2
63	2	2	2	2	2
64	2	2	2	2	2
65	2	2	2	2	2
66	2	2	2	2	2
67	2	2	2	2	2
68	1	2	2	2	1
69	2	2	2	2	2
70	2	2	2	2	2

71	2	2	2	2	2
72	2	2	2	2	2
73	1	1	1	1	1
74	1	1	1	1	1
75	1	1	1	1	1
76	1	1	1	1	1
77	1	1	1	1	1
78	1	1	1	1	1
79	1	1	1	1	1
80	1	1	1	1	1
81	1	1	1	1	2
82	1	1	1	1	1
83	1	2	2	2	2
84	1	2	2	2	2
85	1	2	2	2	2
86	1	2	2	2	2
87	1	2	2	2	2
88	1	2	2	2	2
89	1	2	2	2	2
90	2	2	2	2	2
91	2	2	2	2	2
92	2	2	2	2	2
93	2	2	2	2	2
94	2	2	2	2	2
95	2	2	2	2	2
96	2	2	2	2	2
97	2	2	2	2	2
98	2	2	2	2	2
99	2	2	2	2	2
100	2	2	2	2	2
